# Initial pH of medium affects organic acids production but do not affect phosphate solubilization

**DOI:** 10.1590/S1517-838246246220131102

**Published:** 2015-06-01

**Authors:** Leandro M. Marra, Silvia M. de Oliveira-Longatti, Cláudio R.F.S. Soares, José M. de Lima, Fabio L. Olivares, Fatima M.S. Moreira

**Affiliations:** 1 Universidade Federal de Lavras, Programa de Pós-Graduação em Ciência do Solo, Departamento de Ciência do Solo, Universidade Federal de Lavras, Lavras, MG, Brasil, Programa de Pós-Graduação em Ciência do Solo, Departamento de Ciência do Solo, Universidade Federal de Lavras, Lavras, MG, Brazil.; 2 Universidade Federal de Lavras, Programa de Pós-Graduação em Microbiologia Agrícola, Departamento de Biologia, Universidade Federal de Lavras, Lavras, MG, Brasil, Programa de Pós-Graduação em Microbiologia Agrícola, Departamento de Biologia, Universidade Federal de Lavras, Lavras, MG, Brazil.; 3 Universidade Federal de Santa Catarina, Departamento de Microbiologia, Imunologia e Parasitologia, Universidade Federal de Santa Catarina, Florianópolis, SC, Brasil, Departamento de Microbiologia, Imunologia e Parasitologia, Universidade Federal de Santa Catarina, Florianópolis, SC, Brazil.; 4 Universidade Estadual do Norte Fluminense, Centro de Biociências e Biotecnologia, Universidade Estadual do Norte Fluminense, Campos dos Goytacazes, RJ, Brasil, Centro de Biociências e Biotecnologia, Universidade Estadual do Norte Fluminense, Campos dos Goytacazes, RJ, Brazil.

**Keywords:** chelation, propionic acid, gluconic acid, tartaric acid, malic acid, tropical soils

## Abstract

The pH of the culture medium directly influences the growth of microorganisms and the chemical processes that they perform. The aim of this study was to assess the influence of the initial pH of the culture medium on the production of 11 low-molecular-weight organic acids and on the solubilization of calcium phosphate by bacteria in growth medium (NBRIP). The following strains isolated from cowpea nodules were studied: UFLA03-08 (*Rhizobium tropici*), UFLA03-09 (*Acinetobacter* sp.), UFLA03-10 (*Paenibacillus kribbensis*), UFLA03-106 (*Paenibacillus kribbensis*) and UFLA03-116 (*Paenibacillus* sp.). The strains UFLA03-08, UFLA03-09, UFLA03-10 and UFLA03-106 solubilized Ca_3_(PO_4_)_2_ in liquid medium regardless of the initial pH, although without a significant difference between the treatments. The production of organic acids by these strains was assessed for all of the initial pH values investigated, and differences between the treatments were observed. Strains UFLA03-09 and UFLA03-10 produced the same acids at different initial pH values in the culture medium. There was no correlation between phosphorus solubilized from Ca_3_(PO_4_)_2_ in NBRIP liquid medium and the concentration of total organic acids at the different initial pH values. Therefore, the initial pH of the culture medium influences the production of organic acids by the strains UFLA03-08, UFLA03-09, UFLA03-10 and UFLA03-106 but it does not affect calcium phosphate solubilization.

## Introduction

Acidity has a direct effect on the activity of the soil microorganisms involved in a variety of processes, including organic matter decomposition, mineralization, immobilization, ammonification, nitrification, volatilization, biological nitrogen fixation and insoluble inorganic phosphate solubilization. Therefore, acidity is a chemical property of soils that plays a central role in agriculture.

Microbial genera reported as P-solubilizer are: *Acinetobacter*, *Bacillus*, *Burkholderia*, *Bradyrhizobium*, *Enterobacter*, *Mesorhizobium*, *Paenibacillus*, *Pantoea agglomerans*, *Pseudomonas*, *Rhizobium*, *Serratia marcescens*, *Penicillium*, *Aspergillus*, *Micromonospora* (Alikhani *et al.*, 2006; El-Tarabily *et al.*, 2008; Farhat *et al.*, 2009; Halder *et al.*, 1990; Hameeda *et al.*, 2008; Marra *et al.*, 2011; Marra *et al.*, 2012; Peix *et al.*, 2001; Richa *et al.*, 2007; Rodríguez and Fraga, 1999; Zeng *et al.*, 2012).

Biological, chemical and physical factors may interfere with the ability of soil microorganisms to solubilize insoluble inorganic phosphates. In many cases, acidification is the main mechanism involved in phosphate solubilization. A significant negative correlation between the pH of the culture medium and phosphate solubilization by several genera and species of microorganisms was demonstrated by diverse authors [(Illmer and Schinner, 1992) -r = −0.49, (Chen *et al.*, 2006) -r = −0.80, and (Marra *et al.*, 2011) -r = −0.89]. For instance, *Arthrobacter* sp. solubilized 519.7 mg P L^−1^ when the pH of the culture medium decreased from 6.8 to 4.9 (Illmer and Schinner, 1992). In contrast, several studies have shown phosphate solubilization without a significant negative correlation with culture medium pH. For example, *Pseudomonas* sp. solubilized 31.0 mg P L^−1^ with no alteration of the culture medium, which was at an initial pH of 6.0 (Hariprasad and Niranjana, 2009). Narsian *et al.* (1995) also reported lack of correlation between pH and Ca_3_(PO_4_)_2_ solubilization by *Aspergillus aculeatus* after a 7 day incubation.

Diverse organic acids are reported in literature as being produced by microorganisms during fermentation. The quantity produced varies according to environmental chemical and physical factors and to the organism genome. Although phosphate solubilization is usually attributed to release of organic acids, few studies have identified and quantified organic acids and defined their role in the solubilization of insoluble phosphates by microorganisms. The organic acids mentioned in literature are: oxalic (pKa = 1.38 ± 0.54), 2-ketogluconic (pKa = 2.10 ± 0.54), maleic (pKa = 2.39 ± 0.25), citric (pKa = 2.93 ± 0.28), tartaric (pKa = 3.07 ± 0.34), fumaric (pKa = 3.15 ± 0.10), gluconic (pKa = 3.35 ± 0.35), malic (pKa = 3.61 ± 0.23), glycolic (pKa = 3.74 ± 0.11), lactic (pKa = 3.91 ± 0.11), succinic (pKa = 4.24 ± 0.17), propionic (pKa = 4.79 ± 0.10), butyric (pKa = 4.76 ± 0.10), acetic (pKa = 4.79 ± 0.10) and isobutyric (pKa = 4.85 ± 0.10) [Akintokun *et al.* (2007); Halder *et al.* (1990); Patel *et al.* (2008); Puente *et al.* (2009); Sperber (1958); Vazquez *et al.* (2000); Yi *et al.* (2008)]. Lower pKa implies in higher dissociation and hence strongest effects in lowering pH.

Phosphate solubilization depends not only on the decrease of the culture medium pH but also on other factors, such as exopolysaccharide production secreted by microorganisms. Under the same culture conditions, *Arthrobacter* sp. solubilized 111.7 mg P L^−1^ as the culture medium pH was lowered from 7.0 to 4.5, whereas *Enterobacter* sp. solubilized 632.6 mg P L^−1^ when the culture medium pH decreased from 7.0 to 4.3 which was due to the larger amount of exopolysaccharide produced by *Enterobacter* sp. Authors suggested that EPS with ability of phosphorus - holding may be a novel important factor in the microbial dissolution of tricalcium phosphate acting synergistically with organic acid (Yi *et al.*, 2008).

Besides promoting a decrease in the pH of the medium, low-molecular-weight organic acids also chelate metals in solution, which increases the phosphorus available to plants. The degree of chelation depends on the type of organic acid involved, the number and proximity of carboxyl groups, the type of metal and the pH of the solution (Jones, 1998).

Only one study (Chaiharn and Lumyong, 2009) has assessed the influence of the initial pH of the growth medium on phosphate solubilization; however, these authors did not assess the production of organic acids.

Marra *et al.* (2012) studied the solubilization ability of 82 strains in three types of phosphates (P, Al and Fe) both in solid (82 strains) and liquid (5 strains) media and verified that Ca phosphates are the most solubilized. They also found that there was a significant negative correlation between the pH of the medium and the amount of soluble phosphorus for CaHPO4 (r = −0.51**) and FePO_4_.2H_2_O (r = −0.28**), a relationship that was not observed for Al(H_2_PO_4_)^3^. The highest solubilization of Ca-phosphate is due to its weaker chemical bound. Besides, Ca is a nutrient required in higher amounts than Fe. Al phosphate has the strongest bound and it is not a nutrient for microorganisms.

Therefore, in this study, we sought to assess the influence of the initial pH of the culture medium on the production of low-molecular-weight organic acids and on the solubilization of calcium phosphate.

## Materials and Methods

### Strains

All the five strains were studied previously regarding their ability to solubilize Ca, Al and Fe-phosphates in solid medium (Marra *et al.*, 2012). Ca, Al and Fe-phosphates solubilization by strains UFLA 03-106 and UFLA 03-116 was also studied in liquid medium (Marra *et al.*, 2012). The strains UFLA 03-08 (*Rhizobium tropici*), UFLA 03-09 (*Acinetobacter* sp.), UFLA 03-10 (*Paenibacillus kribbensis*), UFLA 03-106 (*P. kribbensis*) and UFLA 03-116 (*Paenibacillus* sp.) were individually examined for their ability to grow in medium 79 (Fred and Waksman, 1928) at different initial pH values, specifically, 5.0, 6.0 and 7.0. All strains were isolated from surface disinfected cowpea nodules. The strains UFLA 03-08, UFLA 03-09 and UFLA 03-10 demonstrated efficiency in symbiotic biological nitrogen fixation and solubilization of CaHPO_4_ or FePO_4_.2H_2_O, the UFLA 03-106 strain solubilized CaHPO_4_ and FePO_4_.2H_2_O and the UFLA 03-116 strain served as negative control for calcium phosphate solubilization because it is not able to solubilize Ca-phosphate (Marra *et al.*, 2012). Accession numbers (16S rRNA sequences) of the studied strains in GenBank are: JQ041883 (UFLA03-08); JQ041884 (UFLA03-09, JQ041885 (UFLA03-10), JQ041894 (UFLA 03-106) and JQ041897 (UFLA 03-116). The strains were streaked on plates containing the culture medium with different pH values and incubated at 28 °C for 10 days. At the end of this period, the presence or absence of growth was assessed. The study design was completely randomized and included three replicates.

### Solubilization of calcium phosphate and production of organic acids

Two experiments were performed to verify the ability of the above strains to solubilize insoluble inorganic phosphate from calcium phosphate in National Botanical Research Institute's solid and liquid growth media (NBRIP) (Nautiyal, 1999) containing 10 g L^−1^ glucose, 5 g L^−1^ MgCl_2_.6H_2_O, 0.25 g L^−1^ MgSO_4_.7H_2_O, 0.2 g L^−1^ KCl and 0.1 g L^−1^ (NH_4_)_2_SO_4_. NBRIP medium was supplemented with Ca_3_(PO_4_)_2_ to a final concentration of 1000 mg of phosphorus per L in the solid medium and 100 mg of phosphorus per L in the liquid medium; different initial pH values were adjusted: 5.0, 6.0 and 7.0.

To produce and standardize inocula, the strains were inoculated into liquid medium 79 (Fred and Waksman, 1928) containing 0.5 g L^−1^ K_2_HPO_4_, 0.2 g L^−1^ MgSO_4_.7H_2_O, 0.1 g L^−1^ NaCl, 10.0 g L^−1^ mannitol and 0.4 g L^−1^ yeast extract, pH 6.8. Strains were incubated with shaking (110 rpm) at room temperature under aerobic conditions. Readings were performed periodically on a spectrophotometer at a wavelength of 560 nm until an optical density (OD) of 0.5 was reached, which was equal to approximately 10^8^ cells per mL. A 0.85% saline solution was used to adjust cells to the desired density when the OD exceeded 0.5.

For assessment in solid NBRIP medium, Petri dishes containing NBRIP medium at each initial pH condition were inoculated in quadruplicate with 20 μL aliquots of each culture (strain) at an OD of 0.5. The control treatment consisted of non-inoculated NBRIP medium. The culture dishes were incubated at 28 °C, the diameter of the solubilization halo (translucent area surrounding colonies) was measured at the beginning of solubilization, *i.e.*, at the 3^rd^ day and, after the 15^th^ day incubation, using a digital paquimeter, and the Solubilization Index (SI) expressed as halo diameter (mm) / colony diameter (mm) was calculated (Akintokun *et al.*, 2007). The investigated strains were classified based on their SI as demonstrating low (SI < 2.00), intermediate (2.00 < SI < 4.00) and high (SI > 4.0) solubilization capacities.

For assessment in liquid NBRIP medium, a 1 mL aliquot of culture medium 79 with an OD of 0.5 at 560 nm was inoculated into a 125 mL Erlenmeyer flask containing 50 mL of NBRIP medium at different initial pH values. The flasks were incubated at 28 °C with shaking at 130 rpm for 10 days. Subsequently, the samples were centrifuged (19,187 g for 5 min), and the pH was measured, as well as the amount of soluble phosphorus in the supernatant using the phosphomolybdate method (Murphy and Riley, 1962). In addition, the organic acids produced in the medium were quantified. For each initial pH value, a non-inoculated control was assessed. The ability of each strain to solubilize phosphate was calculated as the difference between the concentration of soluble phosphorus in the culture medium of samples that had been inoculated with bacterial strains and that of the non-inoculated control treatment.

High-performance liquid chromatography (HPLC) (Agilent HP Series 1100) was used to identify and quantify organic acids. Samples were collected, filtered through a 0.45 μm cellulose membrane and injected into a Supelcogel C-610H 9 μm chromatographic column measuring 30 cm × 7.8 mm. The eleven Merck® pro-analysis organic acids that have been reported in the literature as being involved in solubilization were used as analytical standards. The mobile phase was 0.1% H_3_PO_4_ (pH 1.81) with a 0.5 mL min^−1^ flow rate and a 100 μL injection per sample. The method was according manufacturer (SUPELCO/SIGMA ALDRICH) of the column Supelcogel. The acquisition time of the chromatograms was estimated to be 30 min with 30 min intervals between runs. Detection was performed by UV at 210 nm with a diode array detector (DAD). The molecules identified and their typical retention times were as follows: oxalic acid (10.10 min), 2-ketogluconic acid (12.10 min), citric acid (12.40 min), gluconic acid (13.04 min), maleic acid (13.33 min), tartaric acid (13.45 min), malic acid (14.85 min), malonic acid (15.23 min), lactic acid (17.89 min), succinic acid (17.91 min) and propionic acid (25.08 min). The quantification of acids was performed using calibration curves of the standards.

The experiment with NBRIP liquid medium was performed in independent assays for each initial pH value with a completely randomized design and two replicates. The results were evaluated by variance analysis using Sisvar (version 4.6) (Ferreira, 2008), and means were compared using the Scott-Knott test at 5%.

## Results

All strains grew in the culture medium 79 at all of the initial pH values studied. In solid NBRIP medium, the strain UFLA 03-116 did not solubilize Ca_3_(PO_4_)_2_ at any of the initial pH values studied. The other strains did solubilize Ca_3_(PO_4_)_2_ at all pH values and exhibited low SI after a 15-day incubation. The only exception was the strain UFLA 03-08 (*R. tropici*), which demonstrated an intermediate SI at all of the investigated initial pH values (Table 1).

**Table 1:** Solubilization index of Ca_3_(PO_4_)_2_ in solid NBRIP medium with different initial values of pH for strains obtained from nodules of cowpea, after 3 and 15 days of incubation at 28 °C.

Strains	pH 5.0		pH 6.0		pH 7.0	
			
	3 days	15 days	3 days	15 days	3 days	15 days
UFLA 03-08(1)	1.39(6)	2.56	1.42	2.41	1.43	2.47
UFLA 03-09(2)	1.13	1.66	1.34	1.79	1.32	1.66
UFLA 03-10(3)	1.17	1.15	1.13	1.10	1.25	1.12
UFLA 03-106(3)	1.19	1.43	1.17	1.50	1.32	1.68
UFLA 03-116(4)	GNS(5)		GNS		GNS	

(1) *Rhizobium tropici*.

(2) *Acinetobacter* sp.

(3) *Paenibacillus kribbensis*.

(4) *Paenibacillus* sp.

(5) GNS= Grew and did not solubilize.

(6) S.I. = halo diameter (mm) / colony diameter (mm), evaluated after 3 and 15 days of incubation.

**Figure 1:**
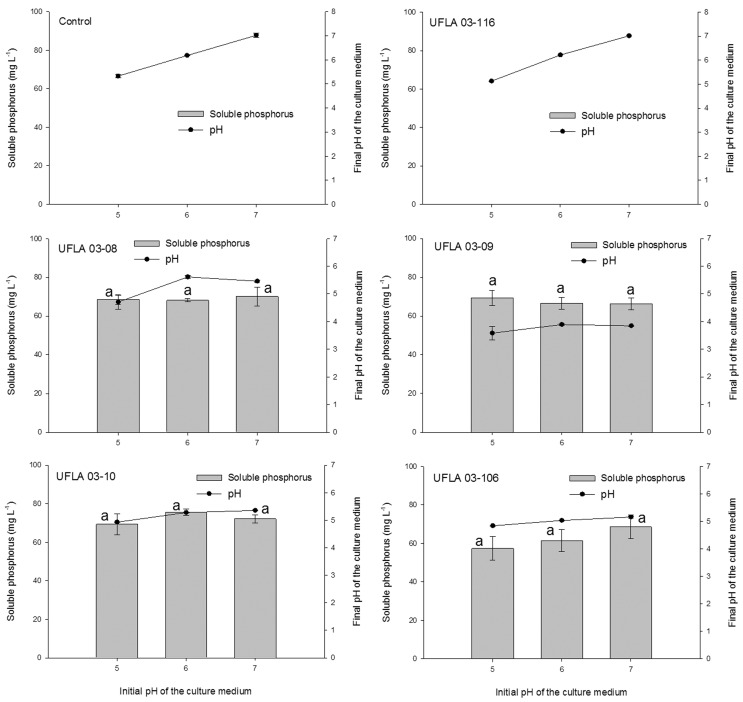
Soluble phosphorus (mg L^−1^) and pH in liquid medium NBRIP after 10 days of incubation with bacterial strains in the presence of Ca_3_(PO_4_)_2_ for different initial values of pH. Error bars represent the standard errors of the means, n = 2.

In liquid NBRIP medium, the strain UFLA 03-116 (*Paenibacillus* sp.) exhibited the same behavior as in the solid medium and did not solubilize Ca_3_(PO_4_)_2_ at any initial pH value, and the pH of the medium did not change from its initial value (Figure 1). The strains UFLA 03-08 (*R. tropici*), UFLA 03-09 (*Acinetobacter* sp.), UFLA 03-10 (*P. kribbensis*) and UFLA 03-106 (*P. kribbensis*) solubilized Ca_3_(PO_4_)_2_ at all initial pH values in liquid NBRIP medium, with no difference being observed in the amount of soluble phosphorus among the different initial pH values of the medium. It is worth noting that more than 60% (as much as 77% in some cases) of insoluble inorganic phosphate (Ca_3_(PO_4_)_2_) was solubilized by these strains.

For most strains, the initial pH of 5.0 did not change after 10-day incubation nor it differed from the control (Figure 1); the only exception was the strain UFLA 03-09 (*Acinetobacter* sp.) in which the pH decreased to less than 4.0. At initial pH values of 6.0 and 7.0, the pH decreased after a10-day incubation among all the strains where solubilization occurred. The greatest difference was found at the initial pH of 7.0, which decreased to less than 4.0 for the UFLA 03-09 strain.

**Figure 2:**
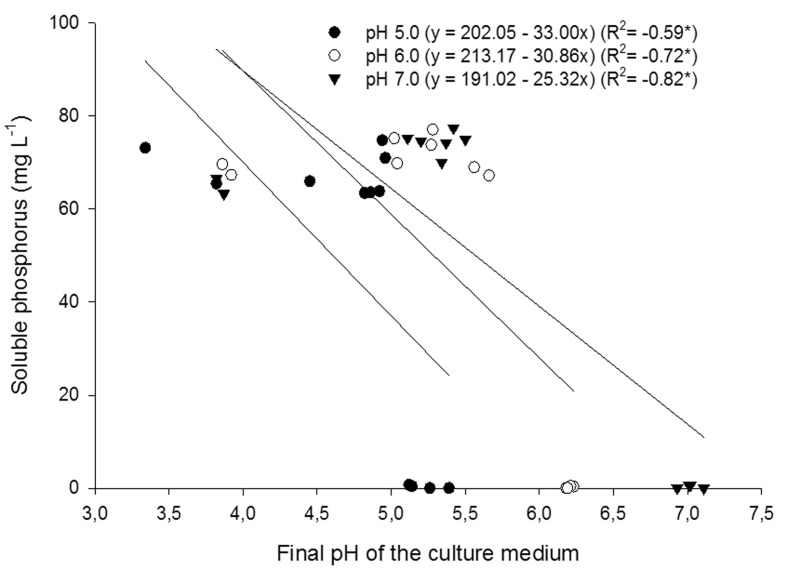
Pearsons correlation between the final pH of the medium NBRIP liquid and the concentration of soluble phosphorus in the presence of Ca_3_(PO_4_)_2_ for different initial values of pH (5.0, 6.0 and 7.0) after 10 days of incubation with strains bacterial (n = 12).

A significant (p < 0.05) negative correlation was found between the amount of soluble phosphorus and the pH of the culture medium at the initial pH of 5.0 (R^2^ = − 0.59), 6.0 (R^2^ = − 0.72) and 7.0 (R^2^ = − 0.82) (Figure 2).

**Figure 3:**
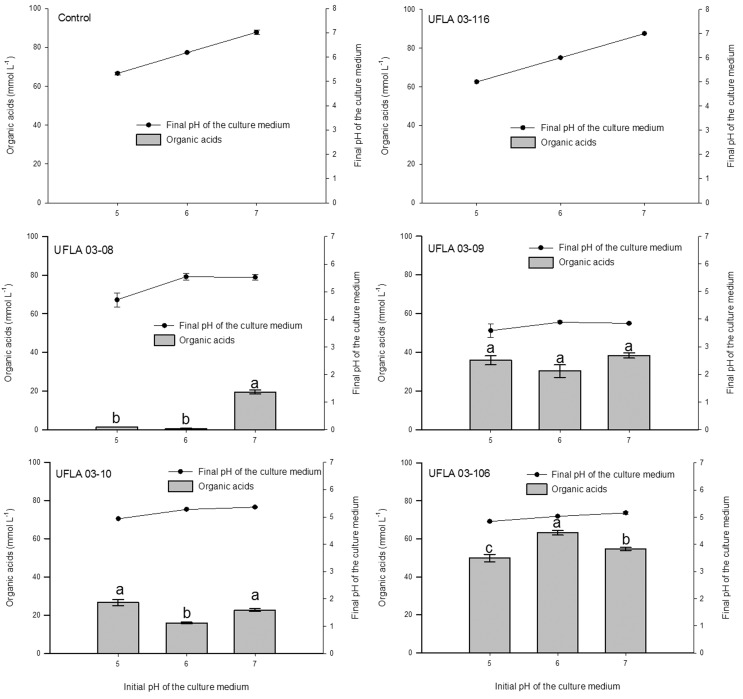
Total organic acids (mmol L^−1^) and pH in liquid NBRIP medium after 10 days of incubation with bacterial strains in the presence of Ca_3_(PO_4_)_2_ for different initial values of pH (5.0, 6.0 and 7.0). Error bars represent the standard errors of the means, n = 2.

With respect to the quantification of organic acids, the strains UFLA 03-08, UFLA 03-09, UFLA 03-10 and UFLA 03-106 produced organic acids in the culture media at all of the initial pH values studied (Figure 3). No organic acids were detected in the control without inoculation and in the negative control, *i.e.*, non-solubilizer strains UFLA 3-116. Considering all of the initial pH values, the highest total acid concentration was produced by the strain UFLA 03-106 (167.7 mmol L^−1^), with the lower concentrations being produced by UFLA 03-09 (104.4 mmol L^−1^), UFLA 03-10 (65.35 mmol L^−1^), and UFLA 03-08 (21.52 mmol L^−1^). Considering all of the strains, the highest total acid concentration was found at pH 7.0 (135.2 mmol L^−1^), with lower concentrations being detected at pH 5.0 (113.65 mmol L^−1^) and pH 6.0 (110.0 mmol L^−1^).

**Figure 4:**
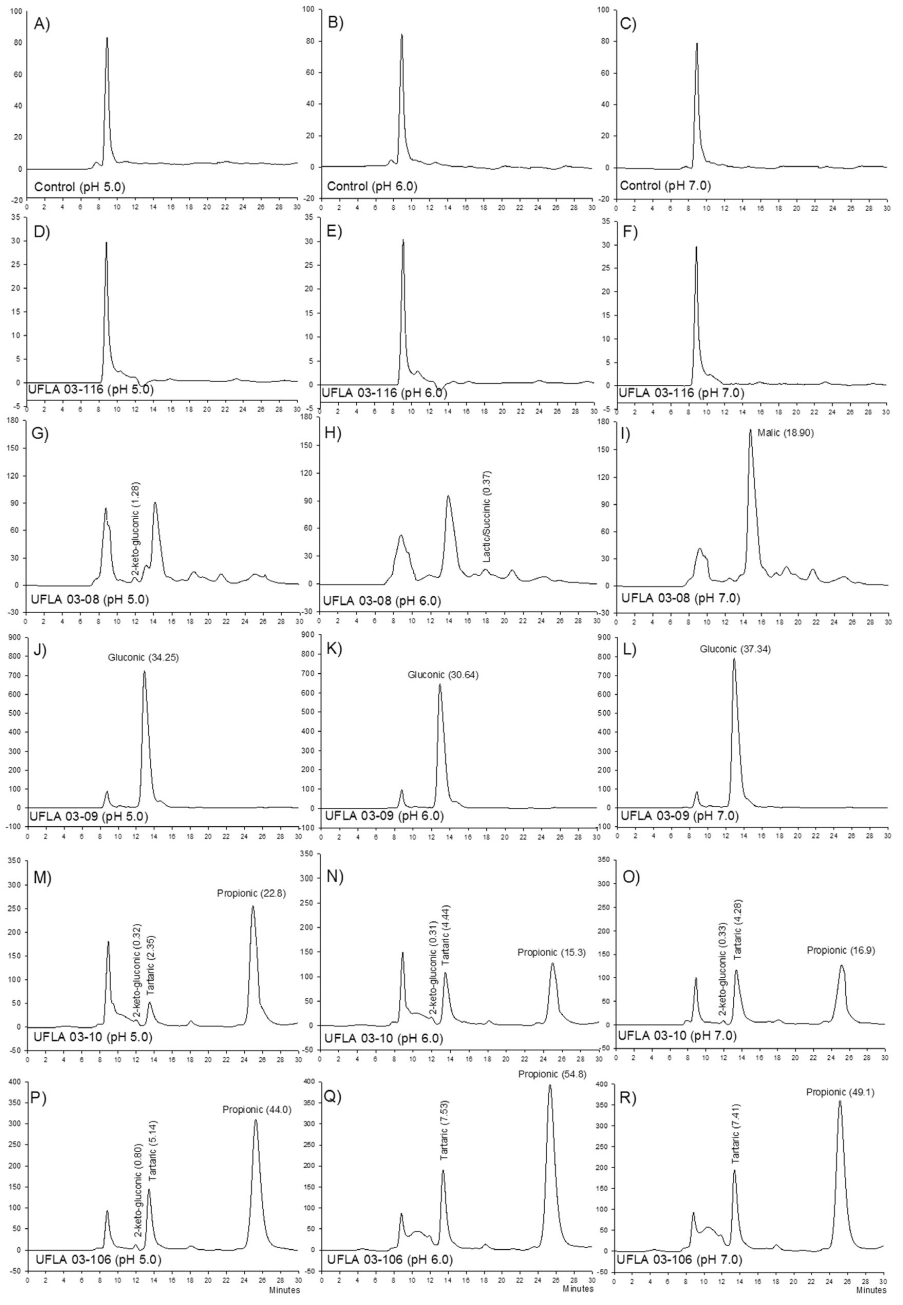
Identification and quantification of organic acids produced in liquid NBRIP culture medium at different initial pH values, inoculated with different bacterial strains. Numbers in parentheses correspond to acid concentration (mmol L^−1^). (Chromatograms - A: control/pH 5.0; B: control/pH 6.0; C: control/pH 7.0; D: UFLA 03-116/pH 5.0; E: UFLA 03-116/pH 6.0; F: UFLA 03-116/pH 7.0; G: UFLA 03-08/pH 5.0; H: UFLA 03-08/pH 6.0; I: UFLA 03-08/pH 7.0; J: UFLA 03-09/pH 5.0 K: UFLA 03-09/pH 6.0; L: UFLA 03-09/pH 7.0; M: UFLA 03-10/pH 5.0; N: UFLA 03-10/pH 6.0; O: UFLA 03-10/pH 7.0; P: UFLA 03-106/pH 5.0; Q: UFLA 03-106/pH 6.0; R: UFLA 03-106/pH 7.0).

With respect to the identification, for the strain UFLA 03-08 (*Rhizobium tropici*), the highest total acid concentration (malic acid, 18.90 mmol L^−1^) was found at an initial pH of 7.0; for this strain, acid production varied with the initial pH of the culture medium. Organic acids identified at pH 5.0 and 6.0 were respectively: 2-ketogluconic (1.28 mmol L^−1^) and lactic/succinic acid (0.37 mmol L^−1^). Other peaks were not identified in all pH values (Figure 4 - Chromatograms G, H and I).

For the strain UFLA 03-09 (*Acinetobacter* sp.), the only acid detected was the gluconic acid, at pH values of 5.0 (34.25 mmol L^−1^), 6.0 (30.64 mmol L^−1^) and 7.0 (37.34 mmol L^−1^), which indicates consistency in the production of acids independent of the initial pH of the growth medium.

The strain UFLA 03-10 (*Paenibacillus kribbensis*) produced 2-ketogluconic (pH 5 = 0.32, pH6 = 0.31, pH7 = 0.33 mmol L^−1^), tartaric (pH 5 = 2.35, pH6 = 4.44, pH7 = 4.28 mmol L^−1^), and propionic acids (pH 5 = 22.8, pH6 = 15.3, pH7 = 16.9 mmol L^−1^) (Figure 4). It can be noticed that the highest total acid concentration was found in the culture media with initial pH values of 5.0 and 7.0 similar to the total quantification (Figures 3). The strain UFLA 03-106, same species of UFLA03-10 (*P. kribbensis*) also produced tartaric (pH5 = 5.14, pH6 = 7.53, pH7 = 7.41 mmol L^−1^) and propionic acid (pH 5 = 44.0, pH6 = 54.8, pH7 = 49.1 mmol L^−1^) at all of the initial pH values but 2-keto-gluconic acid was only detected in pH 5.0 (0.80 mmol L^−1^). For this strain, the highest total acid concentration was found in the medium with the initial pH of 6.0 (Figures 3).

The UFLA 03-116 (*Paenibacillus* sp.) was the only strain that did not produce any organic acids under any culture condition and, therefore, it exhibited the same behavior as the control treatment; the chromatograms show a peak at a retention time of 8.91 min, which differs from all the retention times exhibited by the acids in this study (Figure 4). It is worth noting that this peak was also present in all of the other treatments and does not interfere with the identification and quantification of the acids studied. Under these treatment conditions, citric acid, oxalic acid, maleic acid and malonic acid were not found.

## Discussion

The pH of the culture medium directly influences the growth of microorganisms and the biochemical processes they perform. In many cases, acidification is the main mechanism involved in phosphate solubilization (Halder *et al.*, 1990; Jha *et al.*, 2009; Marra *et al.*, 2011; Marra *et al.*, 2012; Whitelaw, 2000). However, several studies have shown a lack of correlation between solubilized phosphorus and pH of the medium (Chaiharn and Lumyong, 2009; Xie, 2009). Therefore, a better understanding of the behavior of phosphate-solubilizing bacteria inoculated into culture media at different initial pH values may contribute to the production and management of inoculants that improve crop production.

Our results showed that in both solid and liquid NBRIP medium, the initial pH did not affect the solubilizing activity of strain UFLA 03-116 (*Paenibacillus* sp.) because it was not able to solubilize Ca_3_(PO_4_)_2_ under these conditions. These results reveal that the inability to solubilize phosphate under these conditions is intrinsic to this strain, because it grew on solid medium, which was visible in Petri dishes, and liquid medium, as was verified by the presence of bacterial biomass during the centrifugation process. Studies (Marra *et al.*, 2012) performed with this same strain of *Paenibacillus* sp. in solid and liquid GELP medium (Sylvester-Bradley *et al.*, 1982) at an initial pH of 7.0 also demonstrated its inability to solubilize CaHPO_4_, Al(H_2_PO_4_)_3_ and FePO_4_.2H_2_O. The only exception was for FePO_4_.2H_2_O in liquid medium; for this phosphate, more than 20% of the phosphorus was solubilized (Marra *et al.*, 2012). An initial pH of 7.0 in GELP medium may contribute to the solubilization of FePO_4_.2H_2_O by strain UFLA 03-116 (*Paenibacillus* sp.).

With respect to the strains that solubilized Ca_3_(PO_4_)_2_, UFLA 03-08 (*R. tropici*) was the only one to have an intermediate SI, which occurred at all of the initial pH values studied, after a 15-day incubation at 28 °C. These SI values were higher than those reported for *Rhizobium* species obtained from *Crotalaria retusa* and *Crotalaria verrucosa* inoculated onto solid Pikovskaya culture medium (Pikovskaya, 1948) at an initial pH of 7.0 (Sridevi *et al.*, 2007), thereby demonstrating that pH does not interfere with solubilization by this strain.

Conversely, the low SI exhibited by the strains UFLA 03-09, UFLA 03-10 and UFLA 03-106 on solid medium contrasts with that of liquid medium in which these strains solubilized significant quantities of phosphates.

The strains UFLA 03-08 (*R. tropici*), UFLA 03-09 (*Acinetobacter* sp.), UFLA 03-10 (*P. kribbensis*) and UFLA 03-106 (*P. kribbensis*) solubilized Ca_3_(PO_4_)_2_ to a similar degree (more than 60%) at all of the initial pH values of NBRIP medium studied. UFLA 03-09 (*Acinetobacter* sp.) was the only strain that decreased the pH during all treatments, which may be related to its production of gluconic acid. Chaiharn and Lumyong (2009) found that after a 5-day incubation of *Acinetobacter* sp. in nutrient broth with an initial pH of 7.0 or 9.0, the pH of the medium decreased, but the pH increased to 6.17 in medium with an initial pH of 5.0; nevertheless, solubilization of calcium occurred. However, these authors did not assess the production of organic acids.

Several authors have suggested that a decrease in pH due to the production of organic acids and the release of protons is a basic principle of phosphate solubilization, (Chen *et al.*, 2006; Sperber, 1958; Whitelaw, 2000). However, the strains UFLA 03-08, UFLA 03-10 and UFLA 03-106 did not decrease the initial pH of 5.0 after 10-day incubation, thereby demonstrating that acidification is not the mechanism used to promote solubilization at this initial pH, even when organic acids are produced in different concentrations. In this case, the acids may be present in anionic forms and therefore do not function in medium acidification but, rather, in Ca^2+^ chelation (Jones, 1998; Whitelaw, 2000). Moreover, the strain UFLA 03-08 at an initial pH of 6.0 exhibited efficient solubilization, with a decrease of pH but producing a low concentration of lactic/succinic acid (0.37 mmol L^−1^). This result indicates that other solubilization mechanisms are involved and that this strain utilizes different mechanisms when the pH of the medium varies. These mechanisms can be: proton exclusion (via cellular respiration and ammonium absorption as N source) (Illmer P and Schinner F, 1992), siderophores (Hamdali *et al.*, 2008) and exopolisaccharide (EPS) production (Yi *et al.*, 2008). The first two mechanisms were not evaluated in this paper, however, all these three strains produce large amounts of EPS that could act synergistically with acid production as suggested by Yi *et al.* (2008). Non-solubilizer strain UFLA 3-116 also produces large amounts of EPS however it did not produces organic acids.

Conversely, medium acidification occurred at initial pH of 6.0 and 7.0, after a 10-day incubation, followed by the production of lactic/succinic and malic acids by the strain UFLA 03-08, 2-ketogluconic, tartaric and propionic acid by the strain UFLA 03-10, and tartaric and propionic acids by the UFLA 03-106. Propionic acid was produced to the greatest degree by the latter two strains which belong to the same species.

The acids produced in larger amounts (propionic > gluconic > tartaric > malic) have pka varying from 4.79 (propionic) to 3.07 (tartaric) without showing any relationship with phosphate solubilization. On the other hand, strain UFLA03-09 (*Acinetobacter* sp.) which decreased the pH to lower level, only produced gluconic acid which has an intermediate pka (3.65).

The strains UFLA 03-08, UFLA 03-09, UFLA 03-10 and UFLA 03-106 solubilized Ca_3_(PO_4_)_2_ at all three initial pH values studied. Brazilian soils usually exhibit acidic pH values, often varying between 5.0 and 6.5, and the practice of liming aims to reach pH 5.5–6.5. Therefore, these strains may increase and maintain the availability of phosphorus to plants across a wide variety of soil management practices. Besides contributing to solubilization, the production of certain acids by these strains may also serve as a readily accessible source of carbon for these microorganisms (Jones, 1998).

The results show that: the initial pH of the culture medium influences the production of organic acids by the strains UFLA 03-08, UFLA 03-09, UFLA 03-10 and UFLA 03-106 but they do not promote the solubilization of calcium phosphate; thus medium acidification is not the mechanism by which the strains UFLA 03-08, UFLA 03-10 and UFLA 03-106 solubilize calcium phosphate when the initial pH at medium is 5.0 and that strains UFLA 03-09 and UFLA 03-10 produced the same acids when the culture medium exhibited different initial pH values.
